# Smoking has detrimental effects on voice related Quality of Life of University Teachers

**DOI:** 10.12669/pjms.40.8.8631

**Published:** 2024-09

**Authors:** Maham Mehmood, Nazia Mumtaz, Ghulam Saqulain

**Affiliations:** 1 Maham Mehmood, MS (SLP) Speech Language Pathologist, MS (SLP Scholar) Riphah International University, Lahore Pakistan; 2 Nazia Mumtaz, PhD (Rehabilitation Medicine) Head of Department & Professor, Department of Speech Language Pathology, Faculty of Allied health Sciences, Riphah International University, Lahore, Pakistan; 3 Ghulam Saqulain, FCPS (Otorhinolaryngology) Head of Department & Professor, Department of Otorhinolaryngology Capital Hospital PGMI, Islamabad, Pakistan

**Keywords:** Professional Voice Use, Smokers, Teachers, Voice handicap, Voice Quality, Voice Related Quality of Life

## Abstract

**Objective:**

To compare voice related quality of life of smoker and non-smoker university teachers.

**Method:**

This Cross-Sectional descriptive study was conducted at Riphah International University over a period of six months January to June, 2022. A sample of N=352 University teachers of both genders, aged 25 to 65 years, who were faculty members and working at least 8 hours per day in teaching positions with at least one-year experience were included in the study. Demographic sheet, Voice Related Quality of Life (VRQOL) and Voice Handicap Index (VHI) were used for data collection and analysis conducted on SPSS Version 21. Mean scores of VRQOL and VHI for smokers and non-smokers were compared using Mann Whitney U Test. & Spearman’s correlation was utilized to determine any association between the tool scores. P<0.01 was considered significant.

**Results:**

Results reveal that the mean score of Voice related quality of life scale was significantly (p=0.000) higher in smokers compared to non–smokers indicating worse voice quality in smokers. Similarly, voice handicap index scores were much higher in smokers (p=0.000) indicating more handicap in the smokers.

**Conclusion:**

The study concludes that smoking has a detrimental effect on voice and voice related quality of life of university teachers and voice related quality of life as determined by VRQOL scale is significantly better in non-smokers.

## INTRODUCTION

Tobacco smoking has been highlighted as a significant risk factor for health including cardiac, cerebrovascular accidents, pulmonary problems, and cancers involving different organs including larynx as well. It is considered as a leading cause of mortality with around 480,000 deaths per year in United States alone.^1^ Tobacco smoke exposure can result in changes affecting laryngeal mucosa as well as epithelial layer of the vocal folds including inflammation which can lead to voice conditions labelled as dysphonia. It can also lead to pulmonary diseases and increase the risk for infections. The changes in the vocal cord epithelium and laryngeal mucosa can negatively impact vocal function and result in reduced vocal range and irregularity of pitch.^2^

Smoking is a global health issue with around 1.1 billion smokers and 7.7 million deaths due to presence of worldwide smoking as reported in the year 2019^3^, with local prevalence of 21.6% and a higher prevalence in males (36%) compared to females (9%).^4^ Literature reveals a higher prevalence of smoking in teachers of 27%.^5^ The prevalence of voice disorders (VD) is also more common in teachers. A Finnish study by Vertanen-Greis H et al., reported prevalence of 54% among school teachers whereas stress was also identified as another factor which is said to increase risk of voice disorders by 3.6 folds.^6^

A study by Gadepalli C et al., revealed voice difficulties in 30% teachers and 9% non-teachers. Hence, voice difficulties cannot only be attributed to vocal misuse by teachers and similarly teachers are not the only professionals suffering from voice disorders but there are many other factors contributing to voice related complications.^7^ Cigarette smoking is also a risk factor for vocal disorders and cessation of smoking brings positive effects on voice health as well as respiration. It also reduces the risks of future diseases and premature deaths. Apart from other health hazards, smoking also causes chronic respiratory diseases which constitute a global threat to related health factors. About 300 million people suffer from asthma due to smoking and 210 million suffer from chronic respiratory diseases worldwide.^8^ The high prevalence of such an issue is an alarming situation which needs to be catered to urgently. A study by Widuri & Wiratama, reported that smoking affects voice handicap index (VHI) score in active as well as passive smokers with three-fold increased risk of vocal fatigue in active smokers.^9^

The literature reveals that smoking can affect the epithelial lining and micro structures of the vocal ligaments. Higher collagen fiber dispersion is also found in smokers with smoking incriminated to be a leading cause affecting voice acoustics and is responsible for chronic irritation, vocal mass and lower fundamental frequency.^10^

Conventionally smoking related diseases especially pertaining to voice, are frequently identified and diagnosed by health care professionals such as otolaryngologists and speech pathologists. It can affect voice and voice quality, result in vocal polyps, nodules, chronic inflammations and malignancies as well. Voice handicap index (VHI) is often used by speech pathologists to assess voice handicap caused by multiple factors and is a possible indicator for clinical assessment of cases at risk of getting vocal disorders due to smoking.^11^

Keeping in view the hazardous effects and prevalence of smoking, the current study was conceived to cater to the literature gap as regards voice related quality of life (VR-QOL) in university teachers related to smoking with the objective to compare VR-QOL in smokers and non-smoker university teachers. The study may be of significant help to educators and teachers by knowing the effects of smoking on their VR-QOL and Speech pathologists in planning management strategies. It will also serve as a baseline for future research.

## METHODS

This cross-sectional descriptive study was conducted over a period of six months from 1^st^ January, 2022 to 30^th^ June, 2022. A sample of N=352 University teachers was recruited from Riphah International University, University of Management Sciences and Technology (UMT) and University of Central Punjab, utilizing non-probability convenience sampling technique. The sample included university teachers of both genders aged between 25 to 65 years, who were faculty members and were working at least 8 hours per day in teaching positions with minimum one-year experience. The teachers who presented with voice complaints or disorders when they joined university, or had allergy, history of surgery involving larynx or oropharynx and suffering from any laryngeal lesions like vocal nodules and polyps were excluded from the study. A sample of n=357 teachers was calculated utilizing Rao soft online calculator with 95% confidence level and 5% margin of error, however five teachers dropped out of study hence, a sample of N=352 was utilized for analysis.

### Ethical Approval:

It was obtained from Research & Ethics Committee (REC) of Riphah International University vide Ref No REC/RCR &AHS/22/0611 dated 29^th^ December, 2021,

The demographic sheet, Voice Related Quality of Life (VR-QOL) and Voice Handicap Index (VHI) were used to collect data from the selected population. Voice Related Quality of Life (VRQOL)^12^, is a valid 10 item questionnaire which assesses the voice related quality of life using a five points Likert scale. Voice Handicap Index (VHI) is a valid 30 item tool with three domains including Functional, Physical and Emotional and scored on a five points Likert scale with 0 for never and four for always.^13^

### Statistical Analysis:

Following data collection, analysis was done on SPSS Version 21. Descriptive statistics was utilized and categorical variables presented in frequency and percentage, while means were calculated for numerical variables like age, VRQOL and VHI scores. Moreover, the mean scores of VRQOL and VHI were cross tabulated with sociodemographic characteristics and results presented using t-test and Anova statistics. Furthermore, the mean scores of VRQOL and VHI for smokers and non-smokers were compared using Mann Whitney U Test and Spearman’s correlation was utilized to see association between VHI and VRQOL scores and P<0.05 was considered as significant.

## RESULTS

**Fig.1:**
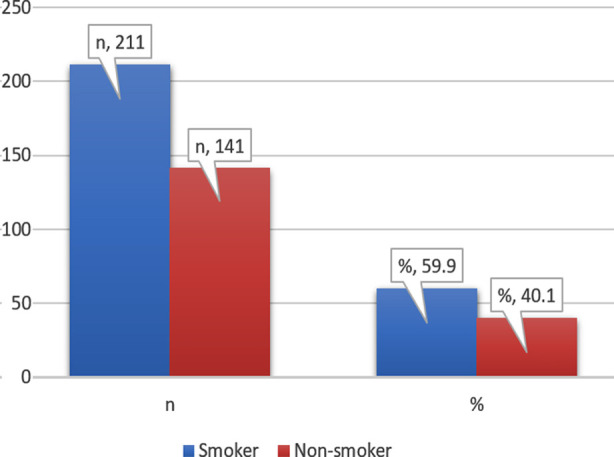
Prevalence of Smoking in University Teachers (n=352).

The current study sample comprised University teachers with mean age of 34.64±6.65 years and a male female ratio of 1.5:1. Study revealed that out of n=352, 211(59.9%) population of university teachers were smokers (Fig.1).

The descriptive statistics for demographic variables and association with VHI and VGRQOL scores is shown in Table-I. Males comprised 211(59.9%) population with all males being smokers and all females being nonsmokers with significant (p<0.001) difference between groups for VHI and VRQOL scores being higher in males.

**Table-I:** Characteristics of Demographic variables & VHI & VRQOL mean scores. Cross tabulation (n=352).

Variable	Group [n(%)]	Smokers (n)		VHI		VRQOL	

		Yes (211)	No (141)	Mean±SD	t/f,p-value	Mean±SD	t/f,p-value
Gender	Male [211(59.9)]	211	0	10.53±4.19	24.01	16.89±1.98	15.62 0.000
	Female [141(40.1)	0	141	0.72±2.98	0.000	14.18±0.70	
Age (Years)	25-40 [289(82.1)	163	126	6.08±6.04	-3.52	15.73±2.08	-1.38 0.169
	46-55 [63(17.9)	48	15	9.02±5.82	0.000	16.13±2.01	
Qualification	MS [300(85.2)]	171	129	6.07±5.92	16.12	15.60±2.01	20.14 0.000
	PHD [52(14.8)]	40	12	9.67±6.29	0.000	16.96±2.10	
Designation/Post	Lecturer [184(52.3)	77	107	4.17±5.22	25.23	15.23±1.60	49.36 0.000
	Senior Lecturer [120(34.1)]	94	26	8.90±6.09	0.000	16.23±2.32	
	Assistant Professor [39(11.1)]	31	8	10.33±5.72		17.10±2.50	
	Associate Professor [9(2.6)]	9	0	9.44±3.36		16.00±1.50	
Teaching/Level	Undergraduate [224(63.6)]	113	111	5.24±5.98	16.88	15.48±1.94	9.56 0.000
	Postgraduate [78(22.2)]	59	19	8.86±5.64	0.000	16.10±1.97	
	Both undergraduate and post graduate [50(14.2)]	39	11	9.20±5.53		16.78±2.45	
Working Hours (per day)	8 [292(83)	164	128	6.01±5.83	-4.13	15.70±2.06	-2.12 0.000
	9 [60(17)]	47	13	9.50±6.60	0.000	16.32±2.09	
Experience Category	2 to 10 [319(90.6)	186	133	6.39±6.08	2.24	15.75±2.00	4.17 0.38
	11 to20 [30(8.5)]	22	8	8.57±6.23	0.108	16.30±2.77	
	>21 [3(0.9)]	3	0	10.00±3.61		16.00±1.73	

Though majority 289(82.1%) were of age group 25-40 years, however higher scores of VHI and VR-QOL were noted for 46-55 years age group with significant p<0.001. difference for VHI scores. Similarly, significantly (p<0.001) higher scores were noted for PhD holders compared to MS degree holders. Though majority were at Lecturer level 184(52.3%), however there was significant difference in VHI and VRQOL mean scores with highest scores for assistant professor level and lowest for lecturers.

Though most of the sample population was teaching undergraduates 224 (63.6), scores of VHI and VRQOL were significantly (p<0.001) higher for those teaching both under graduate and post graduate levels. With mean working hours being 8.17±0.38 hours, majority 292(83%) were working for eight hours a day with significantly (p<0.001) higher VHI and VRQOL scores for those teaching more than eight hours a day. The mean teaching experience was 7.04±3.45 years with most 319 (90.6%) in the experience category of 2-10 years, however mean VHI and VRQOL scores did not reveal any significant difference.

Statistics on normality revealed normal distribution of data with p<0.05, hence necessitating application of non-parametric tests for analysis (Table-II). Mann Whitney U test results applied for comparison between two tool scores. Results reveal that with mean Voice related quality of life scale score was significantly (p=0.000) higher in smokers compared to non –smokers indicating worse voice quality in smokers. Similarly, voice handicap index scores were much higher in smokers (p=0.000) indicating more handicap in smokers (Table-II). Spearman’s rho correlation reveals significant positive correlation between Voices related quality of life scale Score and Voice Handicap Index Score. (r=.794, p=0.000).

**Table-II:** Descriptive Statistics of Normality & Comparison between Groups (n=352).

TESTS FOR NORMALITY OF DATA						

Tool	Kolmogorov-Smirnova			Shapiro-Wilk		

	Statistic	Df	P-value	Statistic	df	P-value
VRQOL TOTAL	0.216	352	0	0.856	352	0.000
VHI TOTAL	0.252	352	0	0.85	352	0.000

***B) MANN WHITNEY U TEST FOR COMPARISON BETWEEN GROUPS***						

***Tool***	***Group***	***Mean±sD***	***Mean Rank***	***Sum of Ranks***	***Z Score***	***P-value***

Voice related quality of life scale	Smoking group	16.88±1.97	237.98	50214	-14.238	0.000
	Nonsmoking group	14.17±0.69	84.50	11914		
Voice handicap index	Smoking group	10.53±4.19	239.51	50537	-14.698	0.000
	Nonsmoking group	0.72±2.98	82.20	11590.5		

**Table-III:** Correlation between Voices related quality of life scale Score & Voice handicap Index Score.

Tool	Spearman’s rho	Voice related quality of life scale	Voice handicap Index
Voice related quality of life scale	R	1	.794**
	P-value	.	0.000
Voice handicap Index	R	.794**	1
	P-value	0.000	.

***(Note:***

** . Correlation is significant at the 0.01 level (2-tailed).

## DISCUSSION

Literature reveals that voice disorders can be caused by pathological changes in larynx and its mechanism, with signs of irritation of larynx including erythema of the vocal cords even in young adults with brief smoking spells.^14^ In current study, to compare the voice related quality of life (VR-QOL) of smoking and non-smoking teachers, a sample with a male female ratio of 1.5:1 and mean age of 34.64±6.65 years was utilized. Most 300 (85.2%) of participants were masters’ degree holders at Lecturer level 184(52.3%) and 224(63.6%) were teaching undergraduates. Their mean working hours were 8.17±0.38 hours and majority 292(83%) working for eight hours a day and mean teaching experience was 7.04±3.45 years with most 319(90.6%) in the experience category of 2-10 years. The study revealed that voice handicap index (VHI) scores were much higher 10.53±4.19 in smokers (p=0.000) indicating more handicap in smokers compared to nonsmokers (0.72±2.98). Similarly, Widuri and Wiratama in their study reported that smoking affected the VHI score in both passive and active smokers.^9^ Even no significant difference was noted between smokers and e-cigarette users in another study.^15^

While Byeon & Cha reported that even the pitch, quality of sound and phonation time are significantly affected by smoking.^16^ Voice disorders are often not considered as serious as other health related problems because they do not directly hint towards mortality but only limits daily living because disrupted voice creates barrier in communication. A study by Merrill RM et al., reported that 38% teachers who were smoking reported limitation in their work because of voice difficulties and 30% experienced unemployment due to voice disorders.^17^ Similarly, the current study results revealed that mean VR-QOL scale score was significantly (p=0.000) higher in smokers (16.89±1.98) compared to non–smokers (14.17±0.69) indicating worse voice quality in smokers. This is also in compliance with study by, Cohen who reported lower voice related quality of life in smokers with dysphonia.^18^

The literature posits that teachers who develop voice disorders also suffer from a poor quality of life (Qol) compared to those who have normal voice.^19^ The current study also revealed a positive correlation between VR-QOL scale score and VHI score. (r=.794, p=0.000). Similarly, a study by Kuntman BD et al., using Turkish VHI and VRQOL reported significant positive correlation between the scores in smokers.^20^ While another study reported that there was moderate correlation between VHI 10 and VRQOL scores among Chinese teachers who had voice issues and those who did not have voice problems.^21^

In current study with a male population of 211 (59.9%) all males were smokers and all females being nonsmokers with significant (p<0.001) difference between groups for VHI and VRQOL scores being higher in males. In contrast, a study by Albustan SA et al. revealed that female teachers were more affected by voice issues compared to males.^22^ The difference is mainly due to the fact that in Pakistani culture smoking is a rare phenomenon in the females. On the other hand, another study reported prevalence of voice problem due to smoking was seen to be equal with respect to both genders. This was due to the similar smoking nature of both male and female in the Western countries.^23^

Sankar G et al., in their study reported that teachers of female gender, those with <10 years’ experience and having to work more than 21 hours in a week showed significant association (p<0.01) with voice issues^24^ In compliance, it was observed in the current study that the teachers who were teaching more than eight hours a day had significantly higher VHI and VRQOL scores. Also, significantly (p<0.001) higher scores of VHI and VRQOL were noted for 46-55 years’ age group for VHI scores compared to other age groups.

A study by Albustan SA et al, revealed that level of teaching also matters with elementary school teachers had significant higher scores compared to middle and high school level in VHI.^23^ Similarly, in the current study though most teachers were teaching undergraduates 224(63.6), however, the scores of VHI and VRQOL were significantly (p<0.001) higher for those teaching students of both under graduate and post graduate levels. Thus, the level of students being taught also impact the voice handicap.^23^

Moreover in the current study significantly (p<0.001) higher scores were noted for PhD holders compared to MS degree holders. Though majority were at Lecturer level 184 (52.3%), however, there was significant difference in VHI and VRQOL mean scores with highest scores for assistant professor level and lowest for the lecturers. These facts point towards the strain on voice organs being the cause of higher VHI and VRQOL scores.

## CONCLUSIONS

The study concludes that smoking has a detrimental effect on voice and voice related quality of life of university teachers and voice related quality of life as determined by VRQOL scale is significantly better in non-smokers.

### Author’s Contribution:

**MM:** Was responsible data collection & analysis & interpretation.

**NM:** Was responsible for conception, methodology and critical revision of the article.

**GS:** Did the writing of manuscript, literature review & responsible for integrity of research and publication of the article.

## References

[1] Centers for Disease Control and Prevention “Health Effects of Cigarette Smoking.”.

[2] Chhabra, Saurabh Kumar, Rakesh Kumar (2012). “Effect of Smoking on Voice and Larynx ”Indian J Otolaryngol Head Neck Surg.

[3] GBD 2019 Tobacco Collaborators (2021). Spatial, temporal, and demographic patterns in prevalence of smoking tobacco use and attributable disease burden in 204 countries and territories, 1990-2019:a systematic analysis from the Global Burden of Disease Study 2019. The Lancet.

[4] Alam S (1998). E. Prevalence and pattern of smoking in Pakistan. J Pak Med Assoc.

[5] Heydari G, Yousefifard M, Hosseini M, Ramezankhani A, Masjedi MR (2013). Cigarette smoking, knowledge, attitude and prediction of smoking between male students, teachers and clergymen in tehran, Iran, 2009. Int J Prev Med.

[6] Vertanen-Greis H, Loyttyniemi E, Uitti J (2020). Voice Disorders are Associated with Stress among Teachers:A Cross-Sectional Study in Finland. J Voice.

[7] Gadepalli C, Fullwood C, Ascott F, Homer JJ (2019). Voice burden in teachers and non-teachers in a UK population:A questionnaire-based survey. Clin Otolaryngol.

[8] Gupta N, Gupta D, Khanna A, Reboucas Filho PP, De Albuguerque YHC (2019). Evolutionary algorithms for automatic lung disease detection. Measurement.

[9] Widuri A, Wiratama E (2021). The Influence of Smoking Habit to Voice Handicap Index Score. Egyptian J Ear, Nose, Throat Allied Sci.

[10] Ayoub MR, Larrouy-Maestri P, Morsomme D (2019). The Effect of Smoking on the Fundamental Frequency of the Speaking Voice. J Voice.

[11] Tafiadis D, Kosma EI, Chronopoulos SK, Papadopoulos A, Drosos K, Siafaka V (2018). Voice Handicap Index and Interpretation of the Cutoff Points Using Receiver Operating Characteristic Curve as Screening for Young Adult Female Smokers. J voice.

[12] Hogikyan ND, Sethuraman G (1999). Validation of an instrument to measure voice-related quality of life (V-RQOL). J Voice.

[13] Barbara H (1997). Jacobson, Alex Johnson, Cynthia Grywalski, Alice Silbergleit, Gary Jaconsen, Michael S. Benninger. The Voice Handicap Index (VHI):Development and Validation. Am J Speech-Language Pathol.

[14] Pinar D, Cincik H, Erkul E, Gungor A (2016). Investigating the Effects of Smoking on Young Adult Male Voice by Using Multidimensional Methods. J Voice Off J Voice Found.

[15] Dealino MA, Dela Cruz APC (2022). Dysphonia in Smokers of Combustible Cigarettes and E-cigarettes Measured Using the Filipino Voice Handicap Index. Philippine J Otolaryngol Head Neck Surg.

[16] Byeon H, Cha S (2020). Evaluating the effects of smoking on the voice and subjective voice problems using a meta-analysis approach. Sci Rep.

[17] Merrill RM, Anderson AE, Sloan A (2011). Quality of life indicators according to voice disorders and voice-related conditions. The Laryngoscope.

[18] Cohen SM (2010). Self-reported impact of dysphonia in a primary care population:an epidemiological study. The Laryngoscope.

[19] Alva A, Machado M, Bhojwani K, Sreedharan S (2017). Study of Risk Factors for Development of Voice Disorders and its Impact on the Quality of Life of School Teachers in Mangalore, India. J Clin Diagn Res.

[20] Kuntman BD, Şahin M, Öğüt MF (2018). Evaluation of the Correlation Between Turkish Voice Handicap Index-10 and Turkish Voice-Related Quality of Life Scale. Turk Arch Otorhinolaryngol.

[21] Lu D, Wen B, Yang H, Chen F, Liu J, Xu Y (2017). A Comparative Study of the VHI-10 and the V-RQOL for Quality of Life Among Chinese Teachers with and Without Voice Disorders. J Voice.

[22] Albustan SA, Marie BS, Naour YS, Darawsheh WB (2018). Kuwaiti Teachers'Perceptions of Voice Handicap. J Voice.

[23] Guimaraes I, Abberton E (2005). Health and voice quality in smokers:an exploratory investigation. Logopedics, phoniatrics, Vocol.

[24] Sankar G, Ganesan V, Shantharam RV, Palanisamy K, Katam I (2022). Epidemiology Of Voice Disorders Among Government School Teachers - An Analytical Cross-Sectional Study from Kancheepuram District. Natl J Community Med.

